# Evidence of biological nitrification inhibition in barley to support sustainable nitrogen management

**DOI:** 10.1093/sumbio/qvag034

**Published:** 2026-08-04

**Authors:** Jack Henderson, Xiaoping Fan, Ellen Elizabeth Smith, Marcel Jaspars, Timothy S George, Cécile Gubry-Rangin

**Affiliations:** School of Biological Sciences, University of Aberdeen, Aberdeen, United Kingdom, AB24 3UU; School of Biological Sciences, University of Aberdeen, Aberdeen, United Kingdom, AB24 3UU; School of Biological Sciences, University of Aberdeen, Aberdeen, United Kingdom, AB24 3UU; Marine Biodiscovery Centre, University of Aberdeen, Aberdeen, United Kingdom, AB24 3UE; International Barley Hub, James Hutton Institute, Invergowrie, United Kingdom, DD2 5DA; School of Biological Sciences, University of Aberdeen, Aberdeen, United Kingdom, AB24 3UU

**Keywords:** BNI, plant-microbe interactions, rhizosphere, microbiome, sustainable agriculture

## Abstract

Nitrification drives nitrogen loss in agricultural systems, resulting in leached nitrate and increased emissions of the greenhouse gas nitrous oxide, thereby reducing nitrogen use efficiency (NUE). Biological nitrification inhibition (BNI) provides a promising nature-based solution to low-NUE by suppressing nitrifying microorganisms via plant-exuded bioactive metabolites. Although BNI is well documented in several major cereal grasses, evidence of BNI in barley (*Hordeum vulgare*) is lacking. This study demonstrates barley BNI activity through suppression of rhizosphere ammonia-oxidizer abundance, without a corresponding inhibition of the total prokaryotic community. Several barley lines exhibited strong inhibition of rhizosphere ammonia oxidizers consistent with high-BNI efficiency. The impact of BNI on the rhizosphere microbial community revealed clear differential effects across multiple phylogenetic clades of ammonia oxidizers, revealing that nitrifier clades differ in sensitivity to BNI in the rhizosphere. This led to a decrease in ammonia-oxidizer community richness correlating with BNI activity. The selective inhibition of rhizosphere ammonia oxidizers suggests that further research across diverse soils is needed. This study clearly demonstrates barley BNI activity in soil through suppression of rhizosphere ammonia oxidizers, identifying promising high-BNI lines, and providing a foundation for developing high-BNI barley cultivars to enhance NUE and increase agricultural sustainability.

**Sustainability Statement**

This study supports multiple United Nations Sustainable Development Goals (SDG), primarily SDG15 (Life on Land), by identifying a sustainable mechanism for enhancing NUE in barley, reducing reliance on nitrogen (N) fertilizer inputs while maintaining productivity and food system resilience. BNI in barley has the potential to reduce nitrate leaching (NO_3_^-^) and nitrous oxide (N_2_O) emissions, contributing to crop sustainability under resource and climate pressures, and thus contributing to SDG2 (Zero Hunger), SDG12 (Responsible Consumption and Production), SDG 6 (Clean Water and Sanitation), SDG 14 (Life Under Water), and SDG 13 (Climate Action).

## Introduction

**Figure 1:**
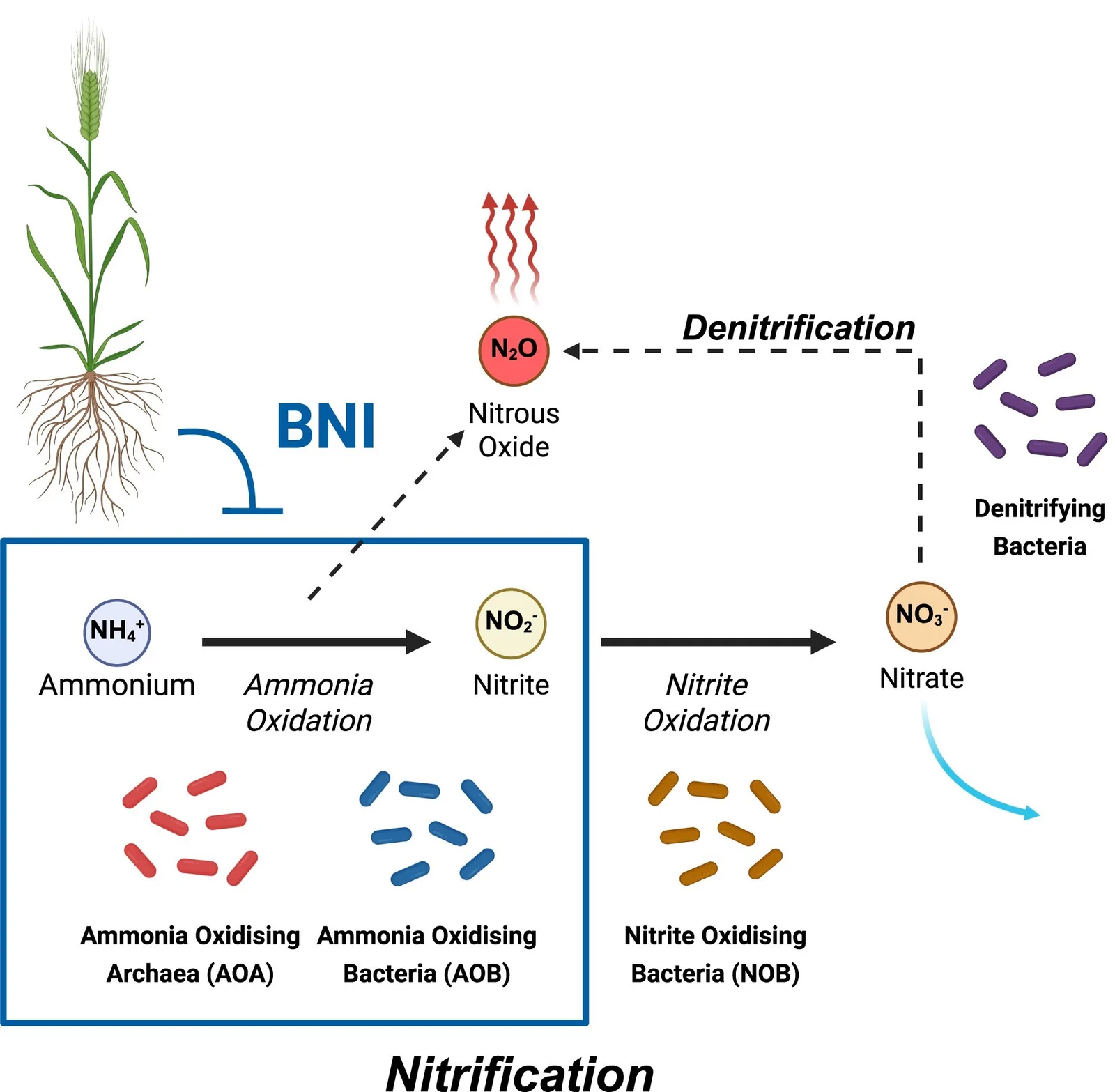
Conceptual diagram of the nitrification–denitrification pathway, illustrating the role of biological nitrification inhibition (BNI) in regulating nitrification via suppression of ammonia oxidizing microbes. The figure was created in https://BioRender.com.

Excessive application of nitrogen (N) fertilizers in modern agriculture drives substantial environmental losses to the atmosphere, through emissions of nitrous oxide (N_2_O), a potent greenhouse gas (GHG) with a global warming potential approximately 300 times stronger than CO_2_ (Griffis et al. 2017), and to water through nitrate (NO_3_^-^) leaching. These losses contribute to low nitrogen use efficiency (NUE) (Menegat et al. 2022, Food and Agriculture Organization of the United Nations 2023, Ludemann et al. 2024). Current global use of N fertilizers exceeds 112 million tonnes per annum, an increase of 32% since 2002, and this is projected to rise by a further 50% by 2050 (Food and Agriculture Organization of the United Nations 2025). The NUE of modern crop systems is approximately 50% (Liu et al. 2025), with the remainder being lost through microbial transformation, primarily nitrification and subsequent denitrification (Fig. 1). Up to ∼60% of global anthropogenic N_2_O emissions are derived from agricultural soils (Smith 2017), thus accounting for approximately 5% of global GHG emissions (CO_2_ equivalent) (Tian et al. 2024; IPCC 2023).

Biological nitrification inhibition (BNI) is the plant-mediated inhibition of nitrification via the exudation of bioactive metabolites (Subbarao et al. 2009), and is a promising emerging strategy for reducing nitrification-driven N loss in agriculture (Fig. 1). Ammonia oxidation is the initial step of nitrification, converting ammonia (NH_3_) into nitrite (NO_2_^-^), via hydroxylamine (NH_2_OH), and is mostly driven by ammonia-oxidizing archaea (AOA) and ammonia-oxidizing bacteria (AOB) in agricultural soils (Gubry-Rangin et al. 2010, Prosser and Nicol 2012). Current evidence suggests that BNI primarily targets this initial step of nitrification (Subbarao et al. 2009, Sun et al. 2016, Bozal-Leorri et al. 2022). The resulting NO_2_^-^ is rapidly oxidized by nitrite-oxidizing bacteria (NOB) to NO_3_^-^, which can then fuel denitrification to N_2_O. In addition to fuelling denitrification, ammonia oxidizers directly emit N_2_O during NH_2_OH oxidation and via nitrifier-mediated denitrification (Prosser et al. 2020). Nature-based solutions such as BNI have the potential to reduce these greenhouse gas emissions and improve NUE.

BNI has been characterized in several major crops within the *Poaceae* family, including rice (*Oryza sativa*) (Tanaka et al. 2010; Sun et al. 2016, Kaur-Bhambra et al. 2023), wheat (*Triticum aestivum*) (O’Sullivan et al. 2017, Subbarao et al. 2021, Jáuregui et al. 2023) and maize (*Zea mays*) (Otaka et al. 2022). BNI activity is driven by the exudation of bioactive metabolites from plant roots, with several factors influencing the observed effect. Previous studies have found that relative BNI activity varies between genotypes within a plant species, with high- and low-BNI lines being identified (Sun et al. 2016, O’Sullivan et al. 2017, Subbarao et al. 2021, Jáuregui et al. 2023, Petroli et al. 2023). High-BNI lines often rely on the exudation of multiple compounds rather than a single compound (Zakir et al. 2008, Subbarao et al. 2013, Sun et al. 2016, Lu et al. 2022, Otaka et al. 2022, Ghatak et al. 2025, Khatri et al. 2026). These BNI compounds exhibit varying efficacy against AOA and AOB, with stronger inhibition of AOA than AOB, and even strain-specific variation in potency (Papadopoulou et al. 2020, Kaur-Bhambra et al. 2022, Kolovou et al. 2023).

While incomplete, there are promising indications that barley (*Hordeum vulgare*) holds strong BNI efficiency, demonstrated by a reduction of gross nitrification rate (GNR) as a proxy for BNI activity (Fountain 2022), with strong genotype dependency among the 200 lines tested. While this study demonstrated substantial variation in soil nitrification rates among barley lines, direct assessment of BNI activity by inhibiting AOA or AOB in soil remains lacking. Therefore, the present study aims to fill this gap and provide substantial evidence of BNI activity in barley by directly assessing inhibition of rhizosphere ammonia oxidizers.

Barley is the fourth highest yielding cereal crop globally (FAOSTAT 2026) and plays a vital economic role, underpinning multi-billion-dollar malting, brewing, and distilling industries. In addition to economic importance, these industries are also culturally significant, shaping regional identities and traditions through products such as beer and whisky, which are deeply embedded in social customs and national identity. Barley is also an important source of animal feed. Improving the NUE of barley production is therefore essential for the long-term economic and environmental sustainability of important global supply chains. Harnessing BNI represents a promising approach to enhance NUE by reducing fertilizer losses, thereby lowering N₂O emissions and NO_3_^-^ leaching, and contributing to both economic and environmental sustainability in agriculture.

This study aims to demonstrate barley BNI activity in soil by quantifying the inhibition of rhizosphere ammonia oxidizers. In addition, we examine the influence of BNI on the diversity and composition of ammonia oxidizers in the rhizosphere. The following hypotheses were tested: (i) barley inhibits ammonia oxidizers in the rhizosphere, with varying efficiencies between lines; and (ii) inhibition is not uniform across members of the nitrifying communities within AOA and AOB.

## Materials and methods

### Plant materials and growth conditions

A targeted panel of barley lines from the International Barley Hub collection comprising ‘Athos’, ‘Barke’, ’Bere: Unst’, ‘Colada’, ‘Decanter’, ‘Diament’, ‘KWS Sassy’, ‘Laureate’, ‘Optic’ and ‘Westminster’ was selected. All of the selected lines, except Bere: Unst were elite or improved cultivars, that are commercially available and agronomically relevant. The selection of cultivars was informed, in part, by a previous study assessing the variation in GNR between barley lines (Fountain 2022). When using a reduction in GNR as a proxy for BNI activity, this study predicted that Athos, Barke, Colada, and Diament were high-BNI lines, while KWS Sassy, Laureate, Optic and Westminster were medium-low BNI lines. Bere: Unst is an ancient landrace collected from the island of Unst, Shetland, Scotland, and is adapted to thrive in nutrient-poor soils (Martin et al. 2025), which explains its selection for BNI testing.

Seeds were germinated in damp vermiculite in darkness for 3 days at 16°C before being transferred to a controlled-environment growth chamber (25°C day, 22°C night; 16 h photoperiod; 80% relative humidity) for 4 days.

Seven days after germination, one uniform-sized seedling was transplanted into a 1.2 L pot filled with 750 g (dry weight) of 4 mm-sieved sandy loam agricultural soil (pH 6.6; SRUC, Craibstone campus, Scotland, United Kingdom), with four biological replicates per barley line. Soil characteristics were described previously by Kemp et al. (1992). All pots were supplemented with 75 mL of a nutrient solution formulated for barley containing 100 µg N g^-1^ soil as ammonium nitrate (NH₄NO₃). The final nutrient solution contained NH₄NO₃ (2.80 g L⁻¹), MgSO₄ (0.36 g L⁻¹), KH₂PO₄ (0.136 g L⁻¹), FeEDTA (0.037 g L⁻¹), MnCl₂ (1.19 mg L⁻¹), H₃BO₃ (1.42 mg L⁻¹), ZnCl₂ (0.098 mg L⁻¹), CuSO₄ (0.04 mg L⁻¹), Na₂MoO₄ (0.042 mg L⁻¹), and CoCl₂ (0.238 mg L⁻¹).

After transplanting, the plants were grown for 28 days in a growth chamber (Snijders Labs Economic Lux) with the above-described conditions. Soil moisture was maintained at 50% water-holding capacity with regular watering, and the pot arrangement was randomized every 2 days. Unplanted pots (*n* = 4), identical to treatment pots, were maintained throughout the experiment as a negative control.

### Plant and soil sampling

The plants were harvested at the late tillering stage (GS25–GS29), as this is a period of high N demand (Bingham et al. 2006) and is associated with increased root exudation in barley (Suku et al. 2014). The plants were carefully removed from the pots and the roots were gently shaken to remove loosely adhering soil, the remaining tightly adhering soil was collected and designated as rhizosphere soil. After multiple root washes, the root and shoot were separated, and plant biomass was measured by weighing after drying at 60°C for 48 h. Unplanted control pots were harvested by discarding the top 2 cm of soil, which is more susceptible to environmental fluctuations, then homogenizing the remaining soil. This ensured that only sub-surface soil was sampled, improving comparability with rhizosphere samples.

Soil pH was measured in a 1:2.5 soil:water slurry after shaking for 30 min and settling for 2 h, using a calibrated pH meter (FiveEasy pH Sensor, Mettler Toledo). Soil ammonium (NH_4_^+^) and NO_3_^-^ were extracted by mixing 0.5 g of soil with 2.5 mL of 2 M KCl. Concentrations of NH_4_^+^ and NO_3_^-^ were measured as described in Kandeler and Gerber (1988) and Hink et al. (2017), respectively.

### DNA extraction and quantitative PCR

Soil genomic DNA was extracted from 0.25 g of rhizosphere soil and unplanted control soil samples using the DNeasy PowerSoil Pro kit (Qiagen) according to the manufacturer’s protocol. The quantity and quality of extracted DNA were assessed by a NanoDrop spectrophotometer (ND-1000 model), with a QC criterion of 260/280 nm absorbance ratio between 1.8 and 2.0.

Ammonia oxidizer abundance was quantified by qPCR of the ammonia monooxygenase alpha-subunit gene (*amoA*) using specific AOA and AOB primer pairs: crenamoA23F/crenamoA616R (Tourna et al. 2008) and amoA1F/amoA2R (Rotthauwe et al. 1997), respectively. Each 20 µL reaction contained 10 µL 2 × iQ SYBR Green Supermix (BioRad), 0.8 µL forward primer (10 µM) and 0.8 µL reverse primer (10 µM; final concentration 0.4 µM each), 0.4 µL bovine serum albumin (BSA; 10 mg mL⁻¹), 2 µL template DNA (diluted to approximately 20 ng µL^-1^), and nuclease-free water (6 µL). Cycling conditions of *amoA* gene consisted of 3 min at 95°C, 40 cycles of 15 s at 95°C, 40 s (bacterial *amoA*) or 35 s (archaeal *amoA*) at 58.5°C, 45 s at 72°C, and 8 s at 80°C for fluorescence reading, followed by a melting curve analysis.

Total prokaryotic (bacteria and archaea) abundance was quantified by qPCR of the V4 region of the 16S rRNA gene using the primer pair 515mF/806mR (Walters et al. 2015). Each reaction contained 10 µL 2 × iQ SYBR Green Supermix, 1 µL forward primer (10 µM) and 1 µL reverse primer (10 µM; final concentration 0.5 µM each), 0.4 µL BSA (10 mg mL⁻¹), 2 µL template DNA, and 5.6 µL nuclease-free water. The cycling conditions were 3 min at 95°C, followed by 40 cycles of 95°C for 15 s, 58.5°C for 35 s, 72°C for 45 s, and 80°C for 8 s (fluorescence measured). A standard curve was run for each target gene, comprising 10-fold dilutions from 10^8^ to 10^1^ copies µL^-1^.

For the *amoA* standard curve, qPCR standards for AOA and AOB *amoA* were generated using genomic DNA extracted from *Nitrosotalea sinensis* (Lehtovirta-Morley et al. 2016) and *Nitrosospira multiformis* (Norton et al. 2002), respectively. The 16S rRNA qPCR standards were derived from genomic DNA extracted from *Escherichia coli*, as previously described (Weisburg et al. 1991). All qPCR runs had a standard curve with an R^2^ > 0.98 and an amplification efficiency of 0.92–1.02. To ensure accurate measurement, technical replicates (*n* = 3) were run for each sample, and the median value was taken. All qPCR assays were run on the Eppendorf realplex^2^ Mastercycler, and the data was analysed using the Eppendorf realplex software.

The BNI efficiency of barley lines in soil was inferred by calculating the percentage inhibition of ammonia oxidizer abundance in comparison with the unplanted control as per:


\begin{eqnarray*}
&& \textit{Inhibition}\ \textit{efficiency}\ \left( \% \right) \\&=& \ \left( {\frac{{\textit{Mean}\ \textit{abundanc}{{e}_{\textit{Control}}} - \textit{Abundanc}{{e}_{\textit{Line}}}\ }}{{\textit{Mean}\ \textit{abundanc}{{e}_{\textit{Control}}}}}} \right) \times \ 100
\end{eqnarray*}

where the inhibition efficiency (%) is the percentage suppression of the mean copy number of the target microbial gene (archaeal or bacterial *amoA* or 16S rRNA), *Mean abundance*_Control_ is the mean copy number of the specific gene in the unplanted control (*n* = 4), and *Abundance*_Line_ is the copy number of specific gene in each biological replicate of each barley line. The inhibition efficiency for each barley line was calculated for each biological replicate relative to the unplanted control and is presented as the mean of the four biological replicates.

### Amplicon sequencing

Amplicon sequencing of the AOA *amoA*, AOB *amoA*, and 16S rRNA genes was performed on DNA extracted from rhizosphere and unplanted soils using the same primers as for qPCR. Target genes were amplified with NEB Q5 High-Fidelity DNA Polymerase, and sample-specific 7-bp barcodes were incorporated into the primers for multiplex sequencing. Amplicon sequencing PCRs were performed in 25 µL reactions containing 5 µL 5 × Q5 Reaction Buffer, 5 µL 5 × Q5 High GC Enhancer, 2 µL dNTP mix (10 mM), 1 µL forward primer (10 µM) and 1 µL reverse primer (10 µM) (final concentration 0.4 µM each), 0.25 µL Q5 High-Fidelity DNA Polymerase (New England Biolabs), 2 µL template DNA, and 8.75 µL nuclease-free water. Cycling conditions for both *amoA* targets consisted of 3 min at 98°C, 35 cycles of 15 s at 98°C, 30 s at 55°C, and 45 s at 72°C, followed by a final extension step at 72°C for 5 min. The conditions for 16S rRNA were as follows: 3 min at 98°C, 35 cycles of 30 s at 98°C, 30 s at 52°C, and 45 s at 72°C, followed by a final extension step at 72°C for 5 min. Derived PCR products were purified using AMPure XP magnetic beads per the manufacturer’s protocol and final DNA concentration was measured by Quant-iT PicoGreen (ThermoFisher Scientific). The 16S rRNA gene amplicons were sequenced on an Illumina NovaSeq platform in PE250 mode, generating approximately 60 000 reads per sample. The AOA and AOB *amoA* amplicons were sequenced on an Illumina NextSeq 2000 platform in PE300 mode, also yielding approximately 60 000 reads per sample. Sequencing runs were provided by PersonalBio (Shanghai, China).

### Bioinformatics

The raw amplicon sequences were processed to amplicon sequence variants (ASVs) using the nf-core/ampliseq (version 2.8.0) (Ewels et al. 2020) pipeline with custom parameter sets. Briefly, primers were removed using Cutadapt (version 4.6) (Martin M 2011) and raw reads were truncated to remove low-quality bases. Reads with more than 2 expected errors were filtered out. DADA2 (Callahan et al. 2016) was used for inference of ASVs and paired-read merging. As the AOA *amoA* amplicon is too long for merging, forward and reverse reads were concatenated with a sequence of 10 N bases in the middle (see Aigle et al. 2019). Taxonomic assignment was performed with the DADA2 (version 1.30.0) implementation of the naïve Bayesian classifier method (Wang et al 2007) and a minimum bootstrap value of 80. The databases used for taxonomic assignment were GreenGenes2 (McDonald et al. 2024) for the 16S rRNA gene, a custom AOA *amoA* database (Alves et al. 2018) and a custom AOB *amoA* database (Aigle et al. 2019). After quality filtering, DADA2 yielded 27 002 16S rRNA, 4531 AOA *amoA*, and 3750 AOB *amoA* ASVs. The alpha diversity (Observed richness, Shannon diversity, and Inverse Simpson diversity) was calculated using the phyloseq package (McMurdie and Holmes 2013) within R (version 4.2.0) (R Core Team 2023). Differences in alpha diversity among barley lines were tested using one-way ANOVA. Beta diversity was evaluated using Bray–Curtis dissimilarities. Ordination was performed using principal coordinate analysis (PCoA) and non-metric multidimensional scaling (NMDS). Variation in beta diversity was assessed by permutational multivariate analysis of variance (PERMANOVA) using the vegan package (version 2.6.10) (Dixon 2003).

Differential abundance of ammonia oxidizers in the rhizosphere of each barley line was quantified relative to the unplanted control. Analysis was conducted at the ‘L3’ level for AOA (Alves et al. 2018) and at the ‘cluster’ level for AOB (Aigle et al. 2019). Prior to analysis, phyloseq objects were pre-processed to remove extremely low-abundance taxa (total abundance < 50 reads across all samples) using the microViz R package (version 0.12.7) (Barnett et al. 2021). Differential abundance was assessed using ANCOM-bc (version 2.4.0) (Lin and Peddada 2020) with barley line as the explanatory variable. ASVs detected in fewer than 10% of biological replicate samples were removed. Biological replicate samples with library sizes below 1000 reads were also excluded from the analysis. Structural zeros were detected. Unbinned taxa were included in analysis but removed for plotting. The relationship between the log_2_FC of specific AOA and AOB taxa and BNI efficiency was examined by Pearson correlation.

### Statistical analysis

All statistical analyses were conducted in R, and figures were generated using the ggplot2 package (version 4.0.0) (Wickham 2016). One-tailed *t*-tests comparing the mean inhibition efficiency for each line against zero were performed to determine which lines significantly reduced qPCR-determined ammonia oxidizer abundance relative to the unplanted control. This was also applied to the total prokaryotic community to determine whether the observed BNI effect is specific to ammonia oxidizers. Soil ammonium and nitrate concentrations, soil pH, and plant biomass were analysed separately by one-way ANOVA, with line as the independent variable and each parameter as the dependent variable. Biomass data was log-transformed to satisfy assumptions of homogeneity of variance and normality. All ANOVAs were followed by post hoc analysis using Tukey’s HSD using the emmeans package (version 2.0.0) (Lenth 2025), with means being considered significantly different when *P* < 0.05. The relationship between rhizosphere pH and BNI efficiency was examined by Pearson correlation.

## Results

### Phenotyping the BNI activity of barley lines

The abundance of ammonia oxidizers and total prokaryotes was quantified in the rhizosphere of each barley line and in the unplanted control (Supplementary Fig. S1). Among ammonia oxidizers, AOA were significantly more abundant than AOB (5.3-fold higher; paired *t*-test on log_10_-transformed data, *P* = 2.2 × 10^–16^).

**Figure 2:**
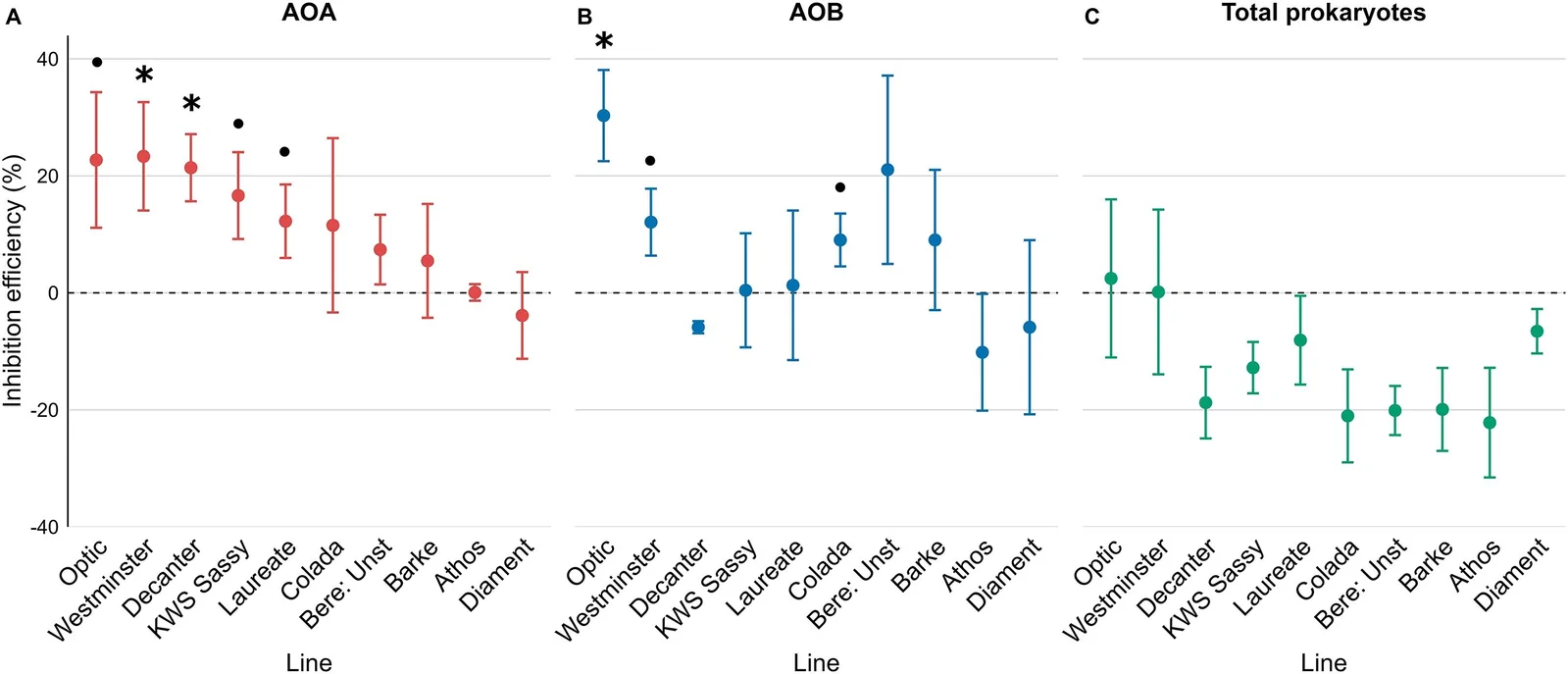
Relative inhibition of (A) AOA, (B) AOB, and (C) total prokaryotes in the rhizosphere of barley lines relative to the unplanted control. Points represent line mean inhibition efficiency (%) and bars indicate ± standard error. One-sample one-tailed *t*-tests determine whether inhibition efficiency of each line is significantly greater than zero (no inhibition), indicating increased BNI activity. Significance levels: *P =* 0.05–0.1 (•), *P* < 0.05 (*).

The BNI efficiency of the barley lines was assessed by their capacity to suppress rhizosphere AOA (Fig. 2A) and AOB (Fig. 2B) relative to the unplanted control. For the purposes of this study, lines exhibiting >20% inhibition were classified as high-BNI, those with <5% inhibition as low-BNI, and intermediate values as medium-BNI. Under this framework, Optic emerges as the foremost high-BNI line, as the only line to show high-BNI activity against both AOA (23%) and AOB (30%). Westminster and Decanter also displayed strong AOA-targeted BNI activity (23% and 21%, respectively), but showed poorer inhibition and, in the case of Decanter, even stimulation of AOB. In Bere: Unst, high inhibition of AOB was observed, although this response was accompanied by substantial variation. Athos and Diament were low-BNI for both AOA and AOB. These categories are intended as a practical guide for informing future studies and current growers seeking to integrate BNI into crop rotations. The current categorizations may not be universal, as relative BNI activity may be influenced by factors such as plant growth stage, soil ammonia-oxidizer community composition, and environmental factors such as soil type, N availability, pH and moisture.

The inhibition observed for the total prokaryotic community did not mirror that of the ammonia oxidizers (Fig. 2C), with no barley lines significantly reducing prokaryotic abundance, even those inhibiting the nitrifiers. Therefore, the nitrifier inhibition observed was likely specific, suggesting the inhibition process is not linked to a general anti-microbial effect.

Despite identifying several promising high-BNI lines, we found that this did not translate into a significant reduction in NO_3_^-^ production between high- and low-BNI lines (Supplementary Fig. S2), indicating that specific differences in BNI activity may not be detectable from end-point NO_3_^-^ measurements alone. Similarly, NH_3_ concentrations did not significantly vary between barley lines. Concentrations of NO_3_^-^ were substantially higher in the unplanted control than in planted treatments, likely due to plant N uptake.

Some lines (Decanter, Westminster) had higher plant biomass than other lines (Supplementary Fig. S3); however, no strong relationship with inhibition efficiency was found. The soil pH of the rhizosphere and the unplanted controls was quantified (Supplementary Fig. S4) and correlations between rhizosphere pH and the inhibition efficiency of AOA and AOB for the barley lines were analysed using Pearson correlation (Supplementary Fig. S5). AOA inhibition decreased significantly with increasing pH (*r* = -0.49, *P* = 1.1 × 10^–3^), whereas AOB inhibition exhibited a non-significant negative trend (*r* = -0.22, *P* = 1.6 × 10^–1^).

### Impact of barley lines on rhizosphere microbiome diversity

**Figure 3:**
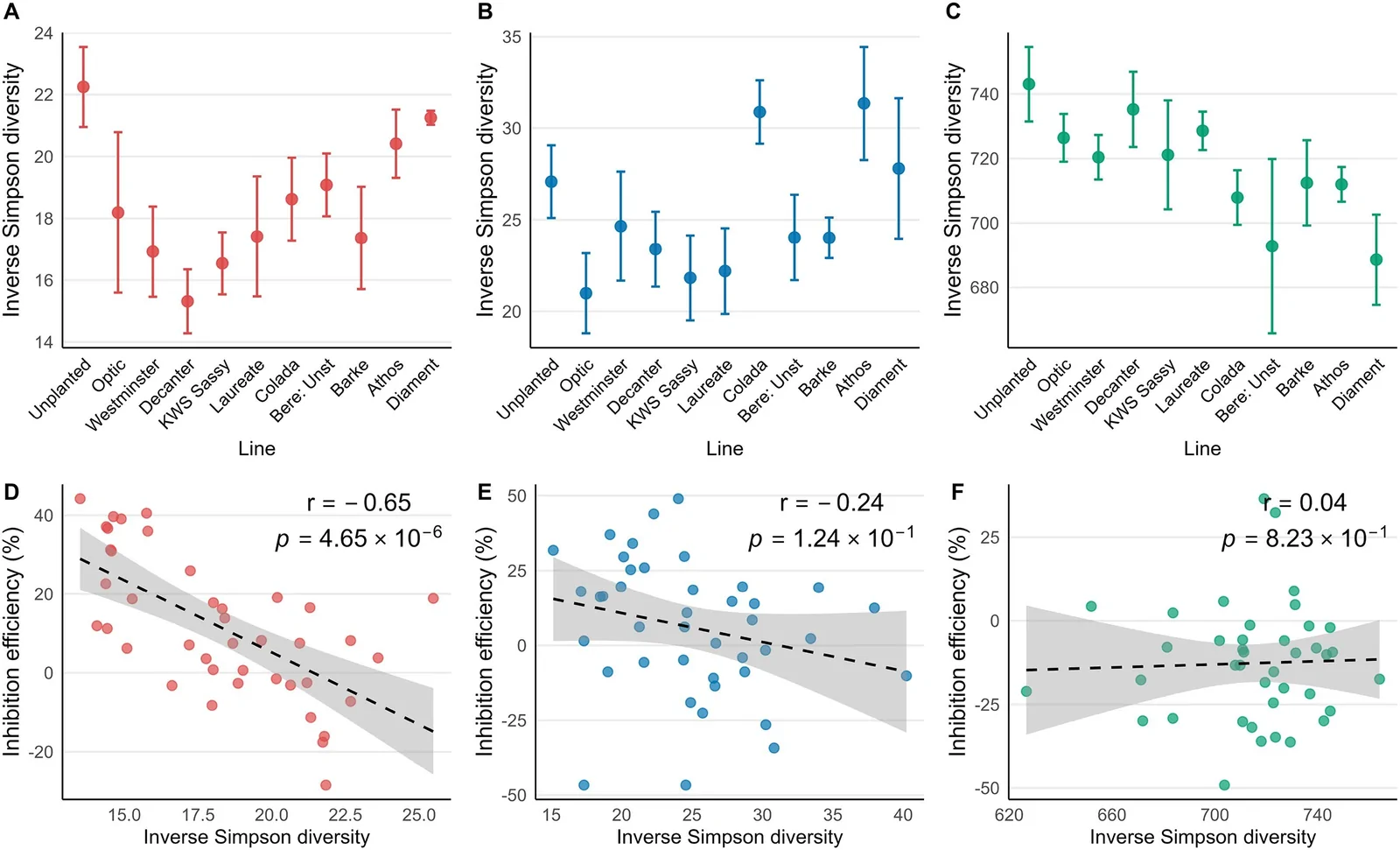
Inverse Simpson diversity of (A) AOA, (B) AOB, and (C) total prokaryotes (16S rRNA) between barley lines and their relationship with inhibition efficiency (D–F). Pearson correlation was used to examine the relationships between (D) AOA inverse Simpson diversity and AOA inhibition efficiency, (E) AOB inverse Simpson diversity and AOB inhibition efficiency, and (E) 16S rRNA inverse Simpson diversity and 16S rRNA inhibition efficiency. All points represent individual biological replicates.

The microbial community composition of AOA (Supplementary Fig. S6), AOB (Supplementary Fig. S7), and total prokaryotes (Supplementary Fig. S8) in the rhizosphere and unplanted soil was elucidated by amplicon sequencing. The microbial alpha-diversity varied between the rhizosphere of the barley lines and the unplanted control. Inverse Simpson diversity (Fig. 3A–C) and Shannon diversity (Supplementary Fig. S9A–C) indicate variation in the richness of abundant and rare microbes, respectively. AOA-based inhibition efficiency showed a strong negative correlation with inverse Simpson (Fig. 3D) (*r* = -0.65, *P* = 4.65 × 10^–6^) and Shannon (Supplementary Fig. S9D) (*r* = -0.70, *P* = 2.85 × 10^–7^) diversities of AOA. Inhibition of AOB displayed the same, though non-significant, trend for both inverse Simpson (Fig. 3E) and Shannon (Supplementary Fig. S9E) diversities. The low-BNI lines Colada, Athos and Diament showed a mean increase in both AOB inverse Simpson (Fig. 3B) and Shannon diversity (Supplementary Fig. S9B) relative to the unplanted control. This pattern suggests that weaker nitrification inhibition may allow a broader range of AOB to persist in the rhizosphere.

Barley line clearly influenced the diversity of the rhizosphere prokaryotic community (Fig. 3C; Supplementary Fig. S9C); however, this variation was not significantly correlated with inhibition. No relationship was observed between inhibition and either inverse Simpson (Fig. 3F; *r* = 0.04, *P* = 0.823) or Shannon diversity indices (Supplementary Fig. S9F; *r* = 0.22, *P* = 0.178).

Analysis of Bray–Curtis dissimilarity (Supplementary Fig. S10) using PERMANOVA reveals that barley line significantly influenced total prokaryotic community composition (F = 1.06, R² = 0.24, *P* = 2.4 × 10^–2^), indicating that even among these closely related barley lines (Schreiber et al. 2024), host identity can shape rhizosphere microbial community structure. Line significantly influenced AOA (F = 1.65, R^2^ = 0.33, *P* = 3.0 × 10^–2^) but not AOB community composition (F = 1.08, R^2^ = 0.25, *P* = 1.7 × 10^–1^), though there was a trend.

### Effect of barley lines on rhizosphere ammonia-oxidizer community composition

**Figure 4:**
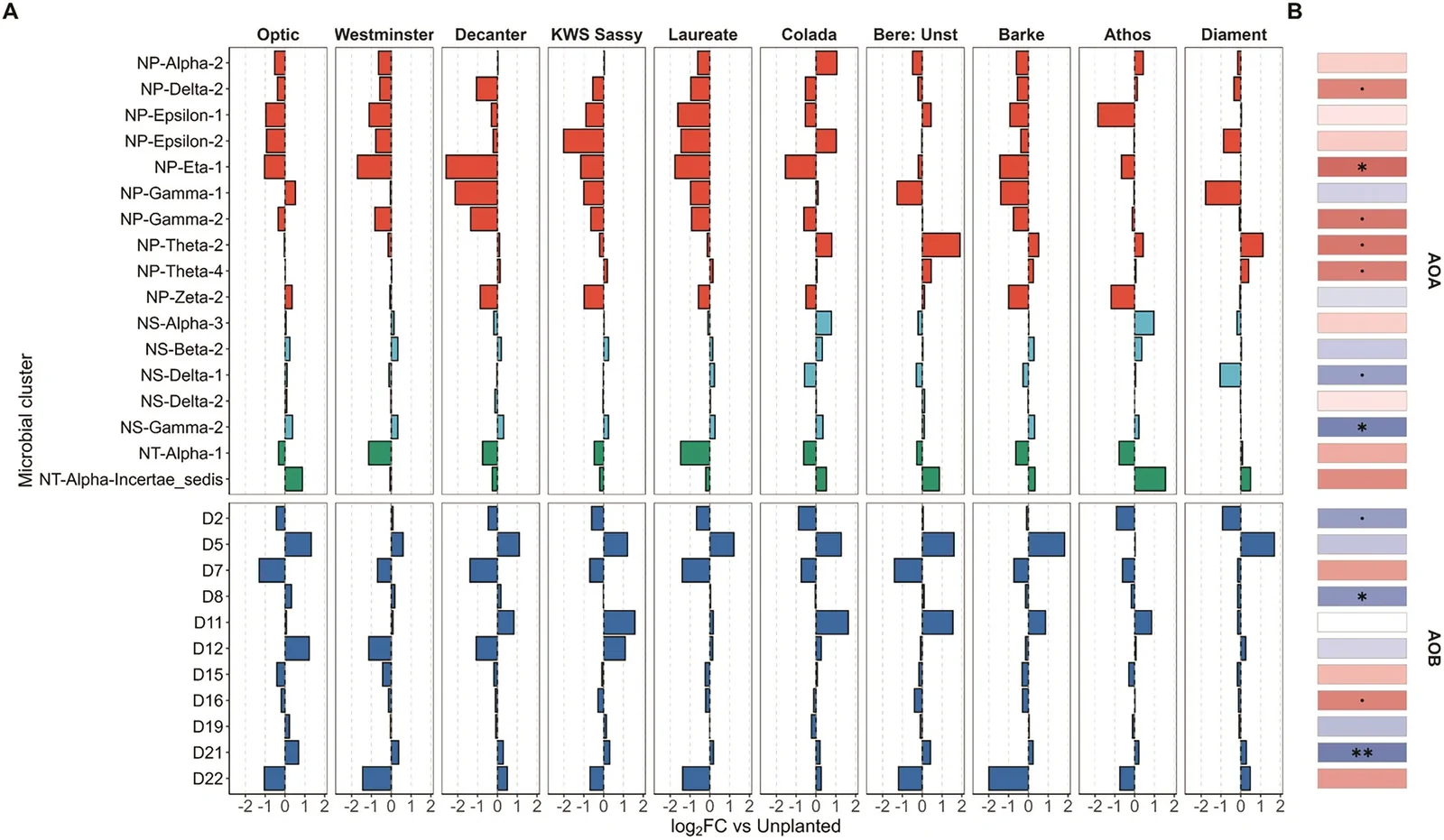
Differential abundance of ammonia oxidizer clusters in the rhizosphere of each barley line, relative to the unplanted control, based on amplicon sequencing of the *amoA* gene. (A) Bars show log₂ fold changes in the relative abundance of individual AOA clusters assigned to *Nitrosopumilales* (NP; red bars), *Nitrososphaerales* (NS; light blue bars) and *Nitrosotaleales* (NT; green bars), and AOB clusters assigned to *Nitrosospira* (D; dark blue bars). Positive values indicate enrichment and negative values indicate depletion relative to the unplanted control. (B) The correlation strip shows Pearson’s *r* between the log₂ fold change of each cluster and the mean inhibition (%) of AOA or AOB across barley lines: positive *r* is represented by blue, while negative *r* is represented by red. AOA log₂ fold change is correlated with AOA inhibition, and AOB log₂ fold change with AOB inhibition. Significance levels: *P* = 0.05–0.1 (•), *P* < 0.05 (*), *P* < 0.01 (**).

Differential abundance analysis of *amoA* sequences revealed taxon-level variation in ammonia-oxidizer community composition between barley lines, with several AOA and AOB taxa showing differential suppression or enrichment in the barley rhizosphere compared to the unplanted control (Fig. 4A).

The AOB *Nitrosospira* D7 cluster was suppressed in the rhizosphere of all lines, but to varying extents, with log₂FC values ranging from -1.40 (Decanter) to -0.64 (Athos). The universal suppression of D7 suggests that this cluster may be particularly sensitive to the selective pressures in the rhizosphere. However, no significant correlation between the log₂ fold change of each cluster and its mean inhibition across barley lines was observed (Fig. 4B); D7 was strongly inhibited by both high-BNI lines, such as Optic (-1.33 log₂FC), and low-BNI lines, such as Decanter. As such, inhibition of this specific ammonia oxidizer cluster alone is not an indicator of overall BNI efficiency. Despite showing relatively low log₂FC between lines, the D16 cluster showed a strong, though non-significant, negative trend with AOB inhibition efficiency (Fig. 4B; *r* = -0.56, *P* = 9.1 × 10^–1^), suggesting that inhibition of this line may contribute to the observed BNI efficiency. This is notable, as D16 was the most relative abundant AOB cluster across all lines (Supplementary Fig. S11). Conversely, D5 was enriched in 90% of barley lines, suggesting this cluster is potentially resistant to barley exuded BNI compounds. Other *Nitrosospira* clusters demonstrate variable effects between barley lines, including D8 and D21, which are significantly correlated (positive log_2_ fold change) with enrichment in high-BNI lines (*r =* 0.64, *P* = 4.5 × 10^–2^ and *r =* 0.78, *P* = 8.0 × 10^–3^, respectively) (Fig. 4B).

AOA also displayed differential sensitivity to barley rhizosphere effects. Several clusters showed significant suppression or enrichment relative to the unplanted control. In general, members of the order *Nitrosopumilales* (NP) were strongly inhibited in the barley rhizosphere, although the degree of inhibition varied among barley lines. For example, NP-Eta-1, which comprised approximately 20% of the AOA community in the unplanted control and the low-BNI line Diament (Supplementary Fig. S12), was consistently suppressed (Fig. 4A), and its log fold change in differential abundance was significantly correlated with BNI activity (*r* = −0.68, *P* = 3.1 × 10⁻²) (Fig. 4B). Several other taxa followed the same trend in suppression correlating with BNI efficiency, including NP-Delta-2 (*r* = -0.55, *P* = 0.098) and NP-Gamma-2 (*r* = -0.62, *P* = 0.054).

Inversely, several AOA clusters showed line-specific enrichment, including the *Nitrososphaerales* cluster NS-Gamma-2, which is significantly enriched in high-BNI lines (*r* = 0.74, *P* = 1.4 × 10^–2^) (Fig. 4B). Several suggestive correlations (e.g. clusters NP-Theta-2, *r* = −0.62, *P* = 0.055; NP-Theta-4, *r* = −0.59, *P* = 0.075) also illustrate the differential effects of BNI on ammonia oxidizer taxa (Fig. 4B).

## Discussion

This study demonstrates barley BNI activity in soil through inhibition of rhizosphere ammonia oxidizers and establishes a foundational germplasm of high-BNI lines. This observed variation in BNI efficiency between lines is also found in other BNI-active plants, such as rice, maize, and wheat (Sun et al. 2016, Subbarao et al. 2021, Petroli et al. 2023). Within the tested lines of barley, Optic is the most promising BNI line, showing strong suppression of both AOA (22%) and AOB (30%) (Fig. 2). Other lines are promising for their BNI efficiency potential, including Westminster and Decanter, which showed BNI activity against AOA but less inhibition, and even stimulation (e.g. in Decanter) of AOB. It should be noted that BNI effects are context-dependent and influenced by soil properties (Zeng et al. 2016, Afzal et al. 2020, Egenolf et al. 2021, Rojas-Pinzon et al. 2024), environmental factors (Zhou et al. 2024, Ma et al. 2025), and microbial community composition (Qin et al. 2025), as indicated in the present study. Therefore, classifications of plant lines as ‘high’ or ‘low’ BNI should be interpreted with caution; however, they provide a useful framework for future experimental design and may offer guidance for land management decisions in the short term. Our results did not reveal significant variation in endpoint NO_3_^-^ production among the barley lines (Supplementary Fig. S2). However, endpoint NO_3_⁻ concentration may be a biased proxy for nitrification or BNI activity, as it impacted by multiple nitrogen cycling processes such nitrification, including heterotrophic nitrification, denitrification, plant uptake, and leaching. Consequently, ammonia oxidizer inhibition may not necessarily translate into detectable differences in final NO_3_⁻ accumulation. We used suppression of rhizosphere ammonia oxidizers as an indicator of BNI efficiency; however, functional redundancy within the nitrifier community may have maintained nitrification despite reductions in specific ammonia-oxidizing taxa. This highlights a limitation of using ammonia oxidizer suppression alone to categorize barley lines as high- or low-BNI. Future studies should aim to determine how factors such as plant growth stage, soil microbial community composition, and environmental conditions influence barley BNI activity and whether inhibition of ammonia oxidizers ultimately translates into improved nitrogen use efficiency. Our results expand the range of crop species for which BNI has been discovered and indicates that BNI is a widespread trait in *Poaceae*, highlighting clear opportunities to explore closely related taxa. As the foremost high-BNI line, future screening efforts should focus on cultivars within the Optic pedigree, including parental lines (Comiche, Force and Chad) and derived cultivars such as Class, Drum and Spire (Shaw et al. 2014, Schreiber et al. 2024). Investigating BNI activity across this lineage may help identify heritable components of the trait and support breeding strategies aimed at developing BNI-enabled barley cultivars with improved NUE. The identification of high-BNI activity in elite barley lines is encouraging, as it creates opportunities for near-term exploitation in both commercial production and breeding programmes. Based on phylogeny (Saarela et al. 2018), temperate crop grasses closely related to barley and wheat (*Pooideae* subfamily) such as *Hordeum spontaneum* (wild barley), rye (*Secale cereale*), and oat (*Avena sativa*) should also be screened for high-BNI efficiency. These species are of major agronomic importance: rye and oat are globally cultivated cereals and forage crops (FAOSTAT 2026). A wild relative of wheat, *Leymus racemosus* (wheatgrass), was found to harbour the genetic basis for high-BNI activity, enabling the development of transformed elite wheat cultivars with high-BNI efficiency (Subbarao et al. 2021, Bozal-Leorri et al. 2022). The BNI efficiency of wild barley should also be assessed, as this may hold the genetic origin of barley BNI activity. Demonstrating BNI in these plants could enable the development of BNI-enabled cultivars across major temperate cereals, contributing to more sustainable nitrogen use in agricultural systems.

The observed variation in AOA and AOB inhibition (Fig. 2) indicates differential responses of these groups to BNI. While our data does not directly identify the underlying compounds, this pattern is consistent with previous studies and may suggest that barley BNI activity is mediated by a mixture of BNI compounds with differing effects on ammonia oxidizers (Lu et al. 2022, Otaka et al. 2022, Ghatak et al. 2025). Other plant mesocosms studies focused on brachiaria (Byrnes et al. 2017), sorghum (Sarr et al. 2021) and Australian tussock grass (Zhou et al. 2024) have also found differential suppression of AOA and AOB, often with stronger inhibition of AOA. This finding is mirrored in culture-based (Kaur-Bhambra et al. 2022, Kolovou et al. 2023) and soil microcosm approaches (Lu et al. 2019, Hua et al. 2025).

Most studies of BNI have not analysed the impact of BNI on ammonia-oxidizer community composition in the rhizosphere soil. To extend this understanding, we examined whether the high-BNI barley phenotypes inhibit all clades of ammonia oxidizers equally or whether barley lines exert differential effects on individual taxa. Similarly to the observed variation in relative inhibition of all AOA and AOB, we also observed differential enrichment or suppression of individual clades between barley lines. Our results show that ammonia oxidizers differ in their sensitivity to BNI activity and that BNI is not broad-spectrum inhibition of AOA and AOB, but instead acts selectively, even towards specific clades within each group. This finding is consistent with culture-based studies which found that ammonia oxidizers demonstrated strain-specific variation in BNI susceptibility (Kaur-Bhambra et al. 2022, Kolovou et al. 2023).

We identify several ammonia-oxidizing taxa which seem to be non-inhibited by BNI compounds, being even significantly proportionally enriched with BNI efficiency (Fig. 4B). These taxa include the AOA *Nitrososphaerales* cluster NS-Gamma-2 and the *Nitrosospira* AOB clusters D8 and D21. This suggests that these taxa may hold tolerance or resistance to BNI, allowing them to exploit the niche left by inhibition of susceptible taxa. The existence of BNI-resistant taxa is reinforced by significant positive correlations with BNI efficiency (Fig. 4B). Several taxa showed sensitivity to BNI (Fig. 4B), including the AOA NP-Eta-1 and the AOB cluster D16. Inhibition of these taxa may play a key role in the observed BNI efficiency. Together, these findings indicate that, similarly to several synthetic nitrification inhibitors, the apparent efficiency of BNI may depend partly on the structure of the resident ammonia-oxidizer community, with tolerant taxa potentially moderating the inhibitory effects of plant lines. Whether changes in the differential abundance of ammonia oxidizers between barley lines are driven by changes in BNI exudation or by other shifts in microbial niche conditions remains unknown. The extent to which BNI efficiency of plant lines is dictated by microbial ecology is not well understood and should be explored in a future purpose-designed study. These findings may have important implications for the long-term efficacy of BNI-enabled agriculture, as long-term enrichments of resistant clusters could ultimately diminish the effectiveness of high-BNI cultivars. Mitigation of such an issue could, for example, involve rotating BNI-producing cultivars or crops to improve the agronomic success of BNI implementation. Despite ammonia oxidizers showing differential susceptibility, the overall effect of BNI is a reduction of the total rhizosphere ammonia oxidizer diversity (Fig. 3) consistent with a filtering effect, where strong selective pressures suppress sensitive taxa and enrich tolerant ones, reducing alpha diversity (Freedman and Zak 2015, Hargreaves et al. 2015). The differential effect of BNI efficiency on the rhizosphere microbiome is further reflected in the relatively high AOB inverse Simpson and Shannon diversity observed in the low-BNI lines Colada, Athos and Diament (Fig. 3B), suggesting a reduction in this filtering effect. These results highlight the close relationship between BNI and the microbial ecology of rhizosphere ammonia oxidizers. The link between BNI and alpha-diversity reduction has been shown in other plant systems such as sorghum (Sarr et al. 2020, Qin et al. 2025) and Australian Tussock grass (Zhou et al. 2024). In general, alpha diversity was lower in the rhizosphere than in bulk soil, consistent with previous studies showing that root activity exerts strong selective pressure on the surrounding microbial community (Philippot et al. 2013, Kaur-Bhambra et al. 2023).

This study provides the crucial first evidence of barley BNI activity in soil through suppression of rhizosphere ammonia oxidizers. However, this is not the first indication of barley BNI activity; a previous large-scale screen assessed the BNI efficiency of barley lines in a large screening panel, using gross nitrification rate (GNR) (Fountain 2022)—measured by quantifying the transformation of ¹⁵N-labelled ammonium after 5 days of incubation—as a proxy for BNI activity rather than assessing inhibition of ammonia oxidizers. The findings of the present study were inconsistent with those of the previous study, in which Optic showed poor BNI efficiency while Barke had the highest BNI efficiency. Several factors could explain this discrepancy. We observed greater inhibition of AOA than AOB (Fig. 2), but the GNR approach favours faster-growing AOB due to the short incubation time and fertilizer addition (Prosser and Nicol 2012, Rütting et al. 2021), therefore likely underestimating AOA inhibition. Given that previous studies have found that AOA drives nitrification in some agricultural soils (Leininger et al. 2006), Gubry-Rangin et al. 2010, Huang et al. 2021), quantification of ammonia-oxidizer communities may be important when interpreting BNI activity. In addition to differential inhibition of AOA and AOB, we observed substantial taxon-level variation in response to BNI (Fig. 4A), as well as evidence that some ammonia-oxidizing taxa may be tolerant to, or even stimulated by, high-BNI activity (Fig. 4B). Together, these findings suggest that the relationship between ammonia oxidizer abundance and nitrification rate may depend on the composition of the resident ammonia-oxidizer community. Environmental factors such as soil type, N availability, pH, and moisture, which shape ammonia-oxidizer communities, may therefore influence the observed BNI activity. Future studies should integrate ammonia-oxidizer community analyses with direct measurements of N transformations and plant N uptake, for example, through ¹⁵N tracing, to assess whether BNI activity enhances NUE and determine how taxon-level variation in BNI response influences nitrification outcomes.

This study provides evidence for BNI activity in barley and identifies Optic as the foremost high-BNI line, capable of strongly inhibiting both bacterial and archaeal ammonia oxidizers *in situ*, providing a foundation for future breeding and mechanistic studies. Our findings indicate that BNI efficiency is shaped by microbial ecology: differential modulation of ammonia oxidizer taxa and evidence of lineage-specific resistance suggest that shifts in community structure may influence the apparent BNI performance of cultivars. This study represents an important step towards the development of future high-BNI barley cultivars, capable of increasing NUE and ultimately reducing the climate impact of barley cultivation.

## Supplementary Material

qvag034_Supplemental_File

## Data Availability

The amplicon sequencing data generated during this study have been deposited in the NCBI BioProject database under accession number PRJNA1451061. All other data supporting the findings of this study are available from the corresponding author upon reasonable request.
