# Supplementary material for: Evidence of biological nitrification inhibition in barley to support sustainable nitrogen management
Source: Sustain Microbiol. 2026 Aug 4;3(3):qvag034. doi: 10.1093/sumbio/qvag034 (PMC13520126; doi:10.1093/sumbio/qvag034)
Supplement: Supplementary file 1 [file qvag034_supplemental_file.docx]

**Supplementary information**

Supplementary figure 1. Abundance of ammonia-oxidising archaea (AOA) and bacteria (AOB) and total prokaryotic populations associated with the rhizosphere of each barley line and the uninhibited control. Bars represent mean gene copy number g^-1^ soil (dry weight) for AOB *amoA*, AOA *amoA*, and 16S rRNA gene. Error bars indicate ± standard error.

Supplementary figure 2. Soil ammonium and nitrate concentration (µg N g^-1^ dry soil) associated with the rhizosphere of each barley line and the bulk soil of the unplanted control. Bars represent mean concentration and error bars represent ± standard error.

Supplementary figure 3. Assessment of plant biomass, represented by root biomass, shoot biomass, total biomass, and root:shoot biomass ratio. Bars represent the mean biomass (g) of 4 biological replicates ± standard error. Letters represent significant differences as assessed by one-way ANOVA, in which ‘line’ was the independent variable, and biomass was the dependent variable.

**10**

Supplementary figure 4. Mean pH of rhizosphere soil for each barley line compared to the unplanted control, ± standard error. The unplanted control is shown in grey, and all planted barley lines are shown in green.

Supplementary figure 5. Pearson correlation between rhizosphere pH and the percentage inhibition of (A) AOA and (B) AOB. Each point represents an individual sample, with the dashed line showing the linear regression fit and the shaded area indicating the 95% confidence interval.

Supplementary figure 6. Relative abundance of bacterial and archaeal phyla across barley lines. Stacked bars represent individual samples, grouped by line, with segment heights showing the proportional abundance of each phylum.

Supplementary figure 7. Relative abundance of AOA clusters at L3 across barley lines and the unplanted control. Stacked bars represent individual biological replicates, grouped by line, with segment heights indicating the proportional abundance of each cluster.

Supplementary figure 8. Relative abundance of AOB clusters across barley lines and the unplanted control. Stacked bars represent individual biological replicates, grouped by line, with segment heights indicating the proportional abundance of each cluster.

Supplementary figure 9. Shannon diversity of (A) AOA, (B) AOB and (C) total prokaryotes (16S rRNA) between barley lines and their relationship with inhibition efficiency (D-F). Shannon diversity indices are represented as mean ± standard error. The relationship between barley inhibition efficiency and Shannon diversity for (D) AOA, (E) AOB and (F) 16S rRNA was determined by Pearson correlation. All points represent individual biological replicates.

Supplementary figure 10. Beta-diversity of 16S rRNA, AOA *amoA* and AOB *amoA* ASVs associated with the rhizosphere of the panel of barley lines and the unplanted (uninhibited) control, represented by Bray-Curtis dissimilarity. Each point represents DNA extracted from the rhizosphere of a single barley plant, or bulk soil in the case of the unplanted control.

Supplementary figure 11. Relative abundance (%) of AOB taxa in bulk soil (red) and barley rhizosphere (blue) across barley lines. Points represent mean relative abundance and error bars indicate ± standard error of biological replicates.

Supplementary figure 12. Relative abundance (%) of AOA taxa in bulk soil (red) and barley rhizosphere (blue) across barley lines. Points represent mean relative abundance and error bars indicate ± standard error of biological replicates.
